# The influence of environmental risk factors in the development of ALS in the Mediterranean Island of Cyprus

**DOI:** 10.3389/fneur.2023.1264743

**Published:** 2023-11-23

**Authors:** Ellie Mitsi, Christiana C. Christodoulou, Paschalis Nicolaou, Kyproula Christodoulou, Eleni Zamba-Papanicolaou

**Affiliations:** ^1^Neurogenetics Department, The Cyprus Institute of Neurology and Genetics, Nicosia, Cyprus; ^2^Neuroepidemiology Department, The Cyprus Institute of Neurology and Genetics, Nicosia, Cyprus

**Keywords:** amyotrophic lateral sclerosis, environmental factors, case–control study, epidemiology, Cypriot population, Mediterranean Island

## Abstract

**Introduction:**

Amyotrophic lateral sclerosis (ALS) is a devastating, uniformly lethal degenerative disease of motor neurons, presenting with relentlessly progressive muscle atrophy and weakness. The etiology of ALS remains unexplained for over 85% of all cases, suggesting that besides the genetic basis of the disease, various environmental factors are implicated in the pathogenesis of ALS. This study aimed to investigate the contribution of known environmental risk factors of ALS in the Cypriot population.

**Methods:**

We conducted a case–control study with a total of 56 ALS cases and 56 healthy gender/age-matched controls of Cypriot nationality. Demographic, lifestyle characteristics, medical conditions, and environmental exposures were collected through the use of a detailed questionnaire. Statistical analyses using the R programming language examined the association between the above environmental factors and ALS.

**Results:**

A chi-square test analysis revealed a statistically significant (*p* = 0.000461) difference in smoking status between the two groups. In addition, univariate logistic regression analysis showed a statistically significant association between ALS cases for head trauma/injury (*p* = 0.0398) and exposure to chemicals (*p* = 0.00128), compared to controls.

**Conclusion:**

This case–control investigation has shed some light on the epidemiological data of ALS in Cyprus, by identifying environmental determinants of ALS, such as smoking, head trauma, and chemical exposure, in the Cypriot population.

## Introduction

Amyotrophic lateral sclerosis (ALS), also known as motor neuron disease (MND), is a progressive neuromuscular disease, characterized by degenerative changes in both upper and lower motor neurons (1). ALS is the most common adult motor neuron disease, with an incidence between 0.6 and 3.8 per 100,000 and a prevalence of 4.1–8.4 per 100,000, while most studies have reported an increasing prevalence over the years (2, 3). Particularly, the incidence and prevalence of ALS in Europe range from 2.16 to 3.80 per 100,000 and 5.24 to 7.40, respectively, while the overall crude worldwide ALS incidence is estimated to be 3.92–4.96 per 100,000 (2–5). According to the Global Burden of Disease (GBD) study in 2016, the global prevalence of ALS was estimated at 4.50 per 100,000 people (6). The male-to-female ratio has been estimated at 2:1, considering male gender as a risk factor for ALS (2, 7–9). ALS is categorized into familial (fALS) and sporadic ALS (sALS), with sALS accounting for 85–90% and characterized by late disease onset, and with a mean age of onset at 58–63 years of age (10). Despite the evolution of diagnostic advancements in the identification of ALS-linked genes, more than 90% of sALS patients still have an unexplained etiology. It is believed that sALS is equally influenced by the interaction of multiple genes (polygenic traits) and environmental risk factors (11). Numerous studies have focused on the environmental risk factors that are positively associated with ALS (12–18). However, dietary factors such as vitamin E supplements and polyunsaturated fatty acids consumption, as well as type 2 diabetes, have shown an inverse association with ALS since they were associated with a lower risk of ALS (11, 16, 19).

Therefore, this current case–control study aimed to investigate potential environmental risk factors for ALS in the Cypriot population. To the best of our knowledge, this is the first study conducted in Cyprus exploring detailed information regarding environmental exposure to various factors (such as smoking, exposure to chemicals, radiation, electric injury, and head trauma), medical history, lifestyle, and anthropometric data that could be associated with ALS.

## Materials and methods

### Study design

**Figure 1:**
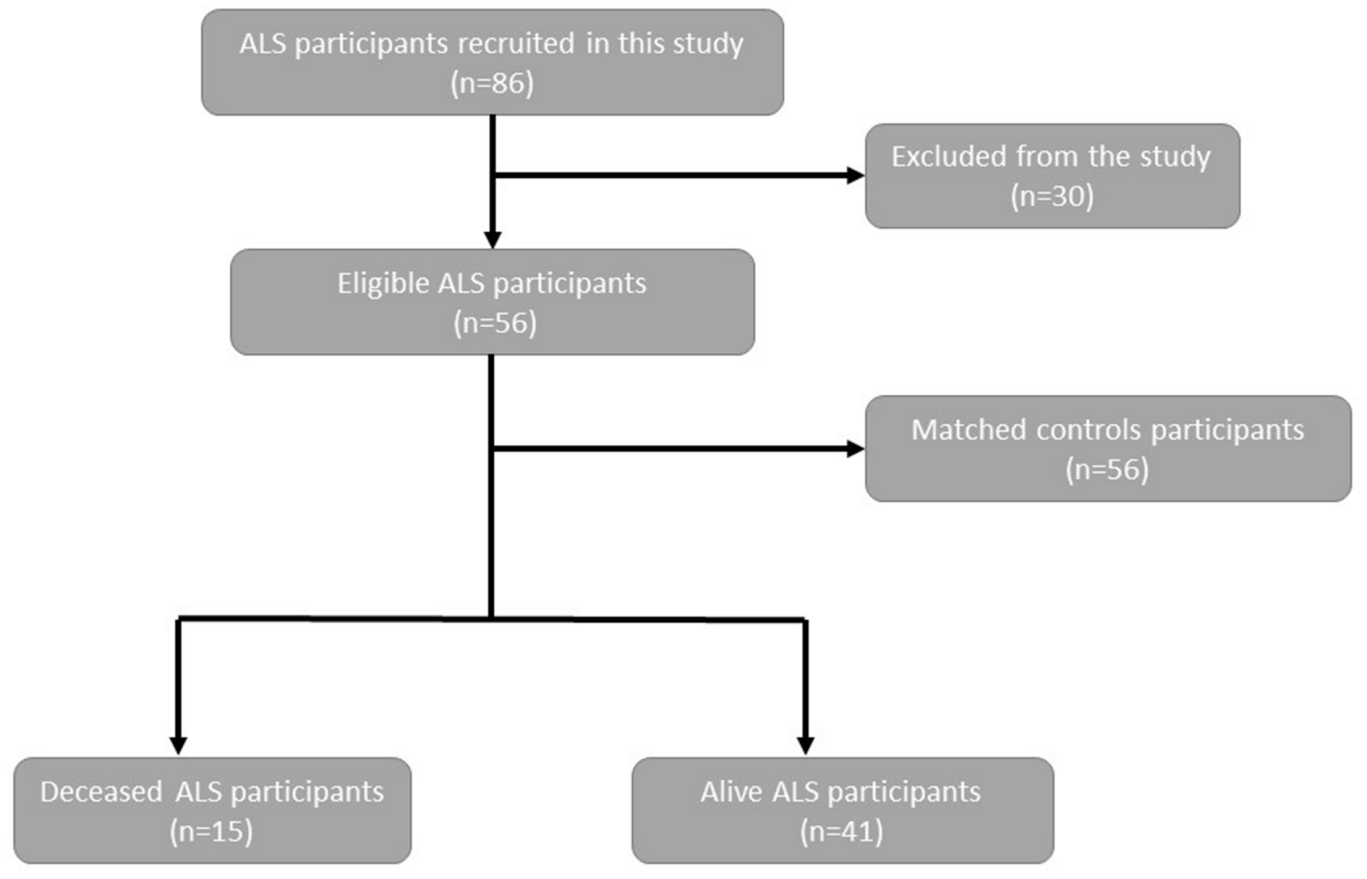
Flow chart for the recruitment of ALS participants and controls in this study.

A total of 86 ALS participants were recruited in this study. Of those, only 56 (41 alive and 15 deceased) were selected according to the inclusion criteria, and their eligibility to fulfill the interview. According to those 56 ALS participants, we recruited an equal number (*n* = 56) of healthy age/gender-matched controls. A total of 112 eligible participants were recruited into the study (Figure 1). All ALS participants being followed up by the Cyprus Institute of Neurology and Genetics (CING), a referral center for ALS in Cyprus, were invited to participate in the study by their clinician. The age of recruitment for ALS cases and controls was between 40 and 89 years old. The inclusion criteria for ALS participants include a (i) definitive diagnosis of ALS according to the El Escorial criteria and (ii) Cypriot nationality. The eligibility criteria for controls included the following: (i) no diagnosis of ALS or other neurodegenerative disease, (ii) no family history of ALS, (iii) Cypriot nationality, (iv) no history of mental disorder, (v) ability to cooperate with the study. All participants were from the area that is under the control of the Republic of Cyprus, and written informed consent was obtained prior to study participation. The study was reviewed and ethically approved by the Cyprus National Bioethics Committee (EEBK/EP/2013/28) and conducted in accordance with the Declaration of Helsinki. Demographic and lifestyle information, including epidemiological data and medical history, was obtained from all study participants through an interviewer-administered questionnaire, through a telephone conversation or via a personal interview. For deceased participants, information was collected from their next of kin, if possible. The information collected is shown in Tables 1, 2.

**Table 1:** Demographics characteristics and smoking status of Cypriot ALS cases and controls.

Variable		Total	Cases	Controls	Value of *p* (test)
Gender					
Male	*N* (%)	56 (50)	28 (50)	28 (50)	1 (chi-square)
Female	*N* (%)	56 (50)	28 (50)	28 (50)	
BMI (kg)					
Underweight <20	*N* (%)	5 (4)	2 (4)	3 (5)	0.29 (chi-square)
Normal 20–24.9	*N* (%)	51 (46)	25 (45)	26 (46)	
Overweight 25–29.9	*N* (%)	34 (30)	21 (38)	13 (23)	
Obesity >30	*N* (%)	22 (20)	8 (14)	14 (25)	
Residence					
Ammochostos	*N* (%)	7 (6)	4 (7)	3 (5)	0.47 (chi-square)
Nicosia	*N* (%)	46 (41)	26 (46)	20 (36)	
Limassol	*N* (%)	51 (46)	20 (36)	31 (55)	
Kerynia	*N* (%)	0 (0)	0 (0)	0 (0)	
Paphos	*N* (%)	5 (4)	3 (5)	2 (4)	
Larnaca	*N* (%)	3 (3)	3 (5)	0 (0)	
Place of birth					
Ammochostos	*N* (%)	29 (25)	15 (27)	14 (25)	
Nicosia	*N* (%)	31 (27)	15 (27)	16 (29)	
Limassol	*N* (%)	30 (26)	13 (23)	17 (30)	0.82 (chi-square)
Kerynia	*N* (%)	11 (10)	7 (13)	4 (7)	
Paphos	*N* (%)	9 (8)	4 (7)	5 (9)	
Larnaca	*N* (%)	2 (4)	2 (4)	0 (0)	
Educational level					
Primary school	*N* (%)	38 (34)	18 (32)	20 (36)	0.82 (chi-square)
Secondary school	*N* (%)	24 (21)	12 (21)	12 (21)	
High school	*N* (%)	26 (23)	15 (27)	11 (20)	
University/Collage	*N* (%)	24 (21)	11 (20)	13 (23)	
Smoking					
Never (No)	*N* (%)	73 (55)	35 (63)	38 (68)	0.000461* (chi-square)
+Ex-Smoker	*N* (%)	6 (11)	6 (11)	–	
∞Ex-Smoker	*N* (%)	6 (11)	6 (11)	–	
Ex-Smoker	*N* (%)	8 (14)	–	8 (14)	
Active (Yes)	*N* (%)	19 (14)	9 (16)	10 (18)	

Significant value of *p* < 0.05, shown in bold*. + Ex-smoker (prior diagnosis). ∞ Ex-smoker (upon diagnosis).

**Table 2:** Reported exposure risk factors in Cypriot ALS cases and controls.

Variables		Total	Cases	Controls	OR* (95% Cl)	*value of p* (LR)**	Value of *p* (LR)***
Head trauma/injury							
Yes	*N* (%)	21 (19)	14 (25)	7 (13)	2.80 (1.08,7.90)	0.0398*	0.035*
No	*N* (%)	91 (81)	42 (75)	49 (88)	1		
Electric injury							
Yes	*N* (%)	16 (14)	13 (23)	3 (5)	5.34 (1.59,24.39)	0.0127*	0.0066*
No	*N* (%)	96 (86)	43 (77)	53 (95)	1		
Exposure to metals							
Yes	*N* (%)	3 (3)	3 (5)	0 (0)	1.28 (0.81,2.05)	0.284	0.29
Yes, work exposure	*N* (%)	24 (76)	14 (25)	10 (18)	1.34 (0.85,2.15)	0.209	0.84
No	*N* (%)	85 (76)	39 (70)	46 (82)	1		
Exposure to chemicals							
Yes	*N* (%)	13 (12)	8 (50)	5 (9)	2.44 (1.46,4.44)	0.00128*	0.00046*
Yes, work exposure	*N* (%)	29 (26)	20 (71)	9 (16)	2.24 (1.39,3.80)	0.00147*	0.0015*
No	*N* (%)	70 (63)	28 (52)	42 (75)	1		
Exposure to radiation							
Yes	*N* (%)	3 (3)	3 (5)	0 (0)	0	0.990	0.08
Yes, work exposure	*N* (%)	2 (2)	1 (2)	1 (2)	1.05 (0.20,5.21)	0.969	0.97
No	*N* (%)	107 (96)	52 (93)	55 (98)	1		
Use of fertilizer							
Yes	*N* (%)	23 (21)	12 (21)	11 (20)	1.14 (0.45, 2.89)	0.777	0.78
Yes, work exposure	*N* (%)	1 (1)	1 (2)	0 (0)	0	0.991	0
No	*N* (%)	88 (79)	43 (77)	45 (80)	1		
Near industrial areas, farms							
Yes	*N* (%)	0 (0)	0 (0)	0 (0)	0		
Yes, work exposure	*N* (%)	15 (13)	9 (16)	6 (100)	1.27 (0.73, 2.28)	0.387	0.390
No	*N* (%)	97 (87)	47 (84)	50 (89)	1		

Significant value of *p* < 0.05, shown in bold*. *Univariate non-adjusted logistic regression model. **p-value nominal significance threshold = 0.05. ***Bonferroni value of p adjusted significance threshold = 0.01.

### Statistical analysis

All statistical analysis was performed using R studio and R statistical packages and scripts, version 4.2.1 (R Core Team, R Foundation for Statistical Computing, Vienna, Austria). The chi-square test (*χ*^2^) and the Mann–Whitney–Wilcoxon test are non-parametric tests that were used to compare the demographic data for ALS cases versus age and gender-matched controls. To estimate the odds ratio (ORs) and 95% CIs for associations between ALS and exposure risk factors in Cypriot ALS cases and age/gender-matched controls, univariate logistic regression analysis was performed using the glm() function in R studio. The level of significance for all tests was defined as a value of p of <0.05. In univariate logistic regression analysis, Bonferroni correction was applied to control the probability of committing type I error (p–value adjusted significance threshold = 0.01).

### Sensitivity analysis

A total number of 56 ALS cases and 56 healthy age/gender-matched controls were initially recruited in this study. However, due to the fact that 15 of those 56 ALS cases were deceased, and information was collected from their next of kin, we excluded 15 controls who were age/gender matched with the deceased ALS cases, who were also excluded, to avoid possible biases. Therefore, statistical analyses of all variables was performed again for confirmational purposes, using 41 alive ALS cases and 41 alive controls. The results of the sensitivity analysis are shown in Supplementary Tables S1, S2.

## Results

### Participants’ characteristics

As shown in Table 1, both cases and controls were equally presented with 50% female patients and 50% male patients, with mean age for both groups estimated at 66.5 years. The mean age of deceased ALS cases was not obtained. The mean age of disease onset was 57 years. Thirteen ALS subjects had an early onset (23.2%), while the remaining 43 participants had a late onset (76.8%). ALS cases were classified according to their initial symptoms into the lumbar onset (89.3%), bulbar onset (8.9%), and frontotemporal dementia–ALS (FTD–ALS; 1.8%). No statistical difference was observed between the two groups regarding educational level (*p* = 0.82), BMI (*p* = 0.29), residence (*p* = 0.47), and place of birth (*p* = 0.82). A statistically significant difference in smoking status was found between cases and controls (*p* = 0.000461). In a total of 21 ALS smokers, only two female cases were reported to have a history of smoking (9.5%), with the remaining 90.5% being male smokers. White-collar occupations were predominant in this study, with controls being more involved in such professions (68%) in comparison to cases (57%). However, no statistically significant association (*p* = 0.33) was observed between the two groups. A number of medical conditions which have been reported by the study population were selected according to prevalence. Both diabetes and high blood pressure were commonly found in controls, with much higher frequency. However, no statistically significant results were found for the medical conditions.

### Risk factors for ALS

**Figure 2:**
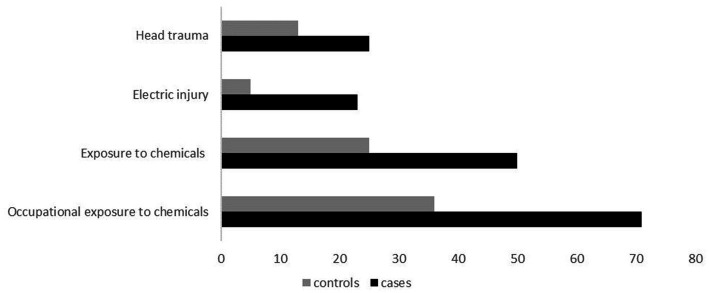
Clustered bar illustration of the most statistically significant variables identified in this study, including head trauma, electric injury, exposure to chemicals, and occupational exposure to chemicals.

As seen in Table 2, there was statistically significant evidence that head trauma/injury, electric injury, and exposure to chemicals, are associated with ALS (shown in Figure 2). Head trauma showed a statistically significant (*p* = 0.0398) difference, exhibiting a high risk of developing ALS if they had a history of head trauma (OR: 2.80, 95% CI: 1.08–7.90). Electric injury (*p* = 0.0127) was also found to correlate with ALS. Cases who have been injured by electric shock at least once in their lifetime have a significantly higher risk of developing ALS (OR: 5.34, 95% CI: 1.59–24.39). Furthermore, given the positive association previously found between lifestyle and/or occupational exposure to chemicals, metals, radiation and fertilizers, and ALS risk, we evaluated this relationship in the Cypriot population. A positive association between exposure to chemical substances (*p* = 0.00128) and risk for ALS was identified between cases and controls (OR: 2.24, 95% CI: 1.46–4.44). Half of ALS cases (28 out of 56) had been exposed to chemical substances, of which 20 cases (71%) reported an occupational exposure (*p* = 0.00147; OR: 2.44, 95% Cl: 1.39–3.80). All of the ALS risk factors remained statistically significant after Bonferroni correction. Nevertheless, no association was observed between the risk of ALS and occupational or lifestyle exposure to metals (*p* = 0,284), radiation (*p* = 0.99), use of fertilizers (*p* = 0.777), and living/working near industrial areas or farms (*p* = 0.387).

### Validation of statistically significant results

Since the above risk factors–smoking, head trauma, electric injury, and exposure to chemicals–were found to be statistically significant in this study, a sensitivity analysis excluding all deceased ALS cases and 15 matched controls was performed. Three of the four previously found significant variables, including smoking, head trauma, and exposure to chemicals, remained statistically significant after performing this analysis. Fertilizers, which were not statistically significant in the initial analysis, showed a value of *p* of 0.0478 (OR: 3.50, 95% Cl: 1.07–13.71) with sensitivity analysis. Additionally, the electric injury was no longer significant (*p* = 0,118; OR: 3.07, 95% Cl: 0.81–14.89), and results were confirmed with Bonferroni correction.

## Discussion

This study investigated environmental exposure risk factors for ALS in Cyprus using detailed epidemiological, demographic, and medical history questionnaires. Interestingly, educational attainment was not correlated with ALS as seen in other studies. For instance, studies have shown that educational attainment is associated with lower odds of ALS (17, 20–22). However, the etiology underlying this association is not yet clear. A possible explanation might be that highly educated individuals are less likely to work and live-in environments exposed to potential risk factors, including chemicals from industrial areas, fuels, fertilizers, and heavy metals. Zhang et al. (23) performed a recent study to investigate the relationship between educational attainment, cognitive-related phenotypes, and ALS. Results were consistent with previous studies, supporting that higher educational attainment conveys a lower risk of developing ALS. This is possibly due to higher cognitive reserve which might be more efficient to offset damages of degenerative brain changes associated with various neurodegenerative diseases including ALS (23). The association between head injury and ALS has also reported by several studies (24–26). In general, patients with ALS have a frequent history of trauma, repeated trauma, and/or severe trauma compared to controls. However, head injury is the most commonly reported type of injury observed in ALS cases (25). A recent meta-analysis study demonstrated that individuals with a history of head trauma had a 1.38-fold increased risk of ALS, supporting that the biological mechanism underlying this association might be the disruption of the blood–brain barrier (24). In our study, ALS cases with a history of head trauma (25%) outnumbered controls (13%). Seven cases with positive head trauma history developed clinical symptoms before the age of 55 years, suggesting that head injury might accelerate ALS disease progression, resulting in an earlier disease onset. However, a limited number of studies have investigated the association of head injury and its role in triggering ALS; therefore, the biological processes remain elusive. Furthermore, the role of BMI in ALS is still unclear, with incompatible results from different epidemiological studies (11, 27, 28). A study had reported a possible positive association of pre-diagnostic BMI with ALS which appeared higher in ALS cases compared to controls (27). On the other hand, Nakken et al. (28) showed that high pre-diagnostic BMI was associated with low ALS risk, indicating a protective role (28). In this current study, even if there is no statistically significant association between BMI and ALS risk, high BMI (25–29.9) was observed in a slightly higher proportion of ALS cases. As BMI increases (>30), the ALS cases decrease significantly with obesity being more prevalent in control groups. These inverse results might demonstrate that metabolic modifications occur several years before the appearance of clinical symptoms in ALS cases.

Smoking is an additional risk factor, and its role in ALS remains unsolved. It is well known that smoking is considered a major health risk for various diseases and conditions, including coronary heart disease, stroke, chronic obstructive pulmonary disease (COPD), and several types of cancer (29). Only in Parkinson’s disease (PD) smoking seems to have an adverse role, with a key role in regulating striatal activity and behaviors mediated through the dopaminergic system since evidence from several epidemiological studies suggests a relationship between cigarette smoking and low risk of PD. (30) However, in ALS cases, toxic substances, including nicotine and formaldehyde, or poisoning contamination by heavy metals and insecticides are a possible explanation of how smoking increases the risk of ALS (17). Results from our study indicate that smoking increases the risk of developing ALS, which comes to agreement with other studies (13, 31–33). A previous ALS epidemiological study in Cyprus by Demetriou et al. (34) found a statistically significant association between male and female cases (34), supporting our outcome with regard to smoking. Other studies report that female smokers have an increased risk of ALS (31) as well as an increased ALS mortality compared to male smokers (35). These results are in opposition to our results, and such inconsistencies are possibly due to limitations related to study design and small sample sizes. Furthermore, a large prospective cohort study by Weisskopf et al., that assessed the association between exposure to chemicals and ALS risk, found that individuals who were exposed to formaldehyde, a substance in cigarette smoke, tend to have an increased risk of ALS (36). However, there are few studies that found no association between smoking and ALS risk, suggesting that cigarette smoking is not considered as a risk factor for ALS (20). Therefore, further observational studies might be required to draw conclusions regarding smoking and ALS risk. According to the literature (21, 37–39), electric shock is also considered a confounding risk factor for ALS. Since 1962, it has been known that electric injury may lead to the development of motor neuron disease (MND) or a syndrome strongly resembling it. This was later confirmed in 1991, in a study by Gallagher et al., which investigated the relationship between electric shock and ALS, where 17 individuals with early-onset ALS had a severe electric injury (37). According to a more recent study, self-reported electrical injury occurring 10 or more years prior to ALS onset was found to increase the risk for ALS (38). Although the exact mechanism by which electric shock could lead to ALS pathology still remains unknown. It is proposed that electric injury could trigger disease onset in individuals at risk of developing the disease by several mechanisms including excitotoxicity and microglial activation (39). Results from our initial analysis showed that electric injury is associated with increased risk for ALS although sensitivity analysis showed no correlation with ALS, indicating that electric injury might not be considered as a risk factor in our population. Exposure to chemicals is another strongly associated risk factor we identified in this study. Half of ALS cases reported long-lasting exposure to various chemicals, whereas the majority of them reported occupational exposure to chemicals. Such occupations fell under the umbrella of blue-collar occupations and mainly include auto-mechanics, builders, landscapers, truck drivers, cleaners, and other working environments that are directly exposed to chemicals. Fertilizers, also considered as chemicals, were previously found to be associated with ALS, particularly in male subjects (20, 40). In our study, a significant association between exposure to fertilizers and ALS was observed after the performance of sensitive analysis, suggesting its possible implication in ALS pathology. However, a higher sample size is required to draw conclusions if fertilizers are indeed a risk factor for ALS. The importance of residence and occupation near industrial areas or farms was also examined. Both cases and controls were near these areas due to their occupations; however, we were unable to identify any significant association between ALS and either exposure variable.

The limitations of this study include those as follows: (i) Small sample size for the analysis of subgroups of certain factors (value of p for medical conditions), which results in low study power. In addition, it is generally adequate for very common risk factors and lacks to provide enough power for the detection of rare exposures. However, Cyprus is a small country, and the recruitment of a larger study population is almost impossible, especially for ALS cases who have lost their mobility and speech. (ii) Recall bias is also a concern when using questionnaires, especially if past or long-term information is required, and participants might be unable to remember potential data. We understand that recall bias is an issue in case–control epidemiological studies, but in this case, due to the nature of ALS and the small sample size contributing to the population of Cyprus, it is unavoidable. (iii) In some cases, family members were not willing to complete the questionnaire, while others were not able to provide information.

To conclude, our study has demonstrated different environmental predictors for ALS in the Cypriot population. Particularly, results support that smoking, head trauma, and exposure to chemicals are indeed risk factors for ALS and were also confirmed with the performance of sensitivity analysis. Other expected factors, including exposure to metals, exposure to radiation, and residence near industrial areas or farms, were found not to be predisposing factors, and neither BMI nor educational attainment were found to be protective for ALS in the Cypriot population. In spite of that, electric injury and fertilizers showed different statistical results with sensitivity analysis. A possible explanation regarding the differentiation between initial statistical analysis and sensitivity analysis is the rapid decrease in sample size, which consequently affects the expected outcomes. Finally, the results of our study highlight the need for further epidemiological studies as well as studies to explore how such factors may potentially influence ALS pathology at the molecular level.

## Data availability statement

The original contributions presented in the study are included in the article/Supplementary material, further inquiries can be directed to the corresponding author.

## Ethics statement

The studies involving humans were approved by the Cyprus National Bioethics Committee (EEBK/EP/2013/28) and conducted in accordance with the Declaration of Helsinki. The studies were conducted in accordance with the local legislation and institutional requirements. The participants provided their written informed consent to participate in this study.

## Author contributions

EM: Conceptualization, Data curation, Formal analysis, Investigation, Methodology, Writing – original draft. CC: Conceptualization, Data curation, Formal analysis, Methodology, Software, Writing – review & editing. PN: Conceptualization, Supervision, Writing – review & editing. KC: Conceptualization, Supervision, Writing – review & editing. EZ-P: Conceptualization, Supervision, Writing – review & editing.
