# Supplementary material for: The influence of environmental risk factors in the development of ALS in the Mediterranean Island of Cyprus
Source: Front Neurol. 2023 Nov 23;14:1264743. doi: 10.3389/fneur.2023.1264743 (PMC10701549; doi:10.3389/fneur.2023.1264743)
Supplement: Supplementary file 1 [file Table_1.DOCX]

***Supplementary Material***

| **Supplementary Table 1:** Sensitivity analysis - Demographics characteristics and smoking status of Cypriot ALS cases and controls. | | | | | |
| --- | --- | --- | --- | --- | --- |
| **Variable** |  | **Total** | **Cases** | **Controls** | ***p-*value (test)** |
| **Gender** |  |  |  |  |  |
| Male | N (%) | 40 (49) | 20 (49) | 20 (49) | 1 (chi-square) |
| Female | N (%) | 52 (51) | 21 (51) | 21 (51) |  |
| **BMI (kg)** |  |  |  |  |  |
| Underweight <20 | N (%) | 5 (6) | 2 (5) | 3 (7) | 0.54 (chi-square) |
| Normal 20-24.9 | N (%) | 32 (39) | 15 (37) | 17 (41) |  |
| Overweight 25-29.9 | N (%) | 28 (34) | 17 (41) | 11 (27) |  |
| Obesity >30 | N (%) | 17 (21) | 7 (17) | 10 (24) |  |
| **Residence** |  |  |  |  |  |
| Ammochostos | N (%) | 6 (7) | 3 (7) | 3 (7) | 0 (chi-square) |
| Nicosia | N (%) | 34 (41) | 20 (49) | 14 (34) |  |
| Limassol | N (%) | 36 (44) | 13 (32) | 26 (56) |  |
| Kerynia | N (%) | 0 (0) | 0 (0) | 0 (0) |  |
| Paphos | N (%) | 4 (5) | 3 (7) | 1 (2) |  |
| Larnaca | N (%) | 2 (2) | 2 (5) | 0 (0) |  |
| **Place of Birth** |  |  |  |  |  |
| Ammochostos | N (%) | 22 (27) | 10 (24) | 12 (29) |  |
| Nicosia | N (%) | 24 (29) | 13 (32) | 11 (27) |  |
| Limassol | N (%) | 19 (23) | 8 (20) | 11 (27) | 0.84 (chi-square) |
| Kerynia | N (%) | 9 (11) | 5 (12) | 4 (10) |  |
| Paphos | N (%) | 7 (9) | 4 (10) | 3 (7) |  |
| Larnaca | N (%) | 1 (1) | 1 (2) | 0 (0) |  |
| **Educational level** |  |  |  |  |  |
| Primary school | N (%) | 26 (32) | 10 (24) | 16 (39) | 0.32 (chi-square) |
| Secondary school | N (%) | 17 (21) | 9 (22) | 8 (20) |  |
| High school | N (%) | 18 (22) | 12 (29) | 6 (15) |  |
| University/Collage | N (%) | 21 (26) | 10 (24) | 11 (27) |  |
| **Smoking** |  |  |  |  |  |
| Never (No) | N (%) | 53 (65) | 26 (63) | 27 (66) | **0.001*** (chi-square) |
| +Ex-Smoker | N (%) | 6 (7) | 6 (15) | 0 (0) |  |
| ∞Ex-Smoker | N (%) | 5 (6) | 5 (6) | 0 (0) |  |
| Ex-Smoker | N (%) | 6 (7) | 0 (0) | 6 (15) |  |
| Active (Yes) | N (%) | 12 (15) | 4 (10) | 8 (20) |  |
| Significant p-value <0.05, shown in **bold***.  + Ex-smoker (Prior Diagnosis). | |  |  |  |  |
| ∞ Ex-smoker (Upon Diagnosis). | |  |  |  |  |
