# Supplementary material for: The influence of environmental risk factors in the development of ALS in the Mediterranean Island of Cyprus
Source: Front Neurol. 2023 Nov 23;14:1264743. doi: 10.3389/fneur.2023.1264743 (PMC10701549; doi:10.3389/fneur.2023.1264743)
Supplement: Supplementary file 2 [file Table_2.DOCX]

***Supplementary Material***

| **Supplementary Table 2:** Sensitivity analysis - Reported exposure risk factors in Cypriot ALS cases and controls. | | | | | | | |
| --- | --- | --- | --- | --- | --- | --- | --- |
| **Variables** |  | **Total** | **Cases** | **Controls** | **OR* (95% Cl)** | ***p-*value (LR)**** | ***p-*value (LR)***** |
| **Head trauma/injury** |  |  |  |  |  |  |  |
| Yes | N (%) | 19 (23) | 15 (37) | 4 (10) | 5.33 (1.71,20.40) | **0.00675*** | **0.0036*** |
| No | N (%) | 63 (77) | 26 (63) | 37 (90) | 1 |  |  |
| **Electric injury** |  |  |  |  |  |  |  |
| Yes | N (%) | 10 (12) | 8 (20) | 2 (5) | 3.07 (0.81,14.89) | 0.118 | 0.11 |
| No | N (%) | 72 (88) | 33 (80) | 39 (95) | 1 |  |  |
| **Exposure to metals** |  |  |  |  |  |  |  |
| Yes | N (%) | 3 (4) | 3 (7) | 0 (0) | 0 | 0,99 | 0,062 |
| Yes, Work Exposure | N (%) | 14 (17) | 9 (22) | 5 (12) | 1.49 (0.83,2.82) | 0,827 | 0,19 |
| No | N (%) | 65 (79) | 29 (71) | 26 (88) | 1 |  |  |
| **Exposure to chemicals** |  |  |  |  |  |  |  |
| Yes | N (%) | 11 (13) | 8 (20) | 3 (7) | 4.91 (1.25, 24.49) | **0.0302*** | **0.021*** |
| Yes, Work Exposure | N (%) | 17 (21) | 14 (34) | 3 (7) | 2.93 (1.56,6.38) | **0.002*** | **0.00048*** |
| No | N (%) | 54 (66) | 19 (46) | 35 (85) | 1 |  |  |
| **Exposure to radiation** |  |  |  |  |  |  |  |
| Yes | N (%) | 3 (4) | 3 (7) | 0 (0) | 0 | 0.99 | 0.079 |
| Yes, Work Exposure | N (%) | 2 (2) | 1 (2) | 1 (2) | 1.03 (0.20,5.29) | 0.957 | 0.96 |
| No | N (%) | 77 (94) | 37 (90) | 40 (98) | 1 |  |  |
| **Use of fertilizer** |  |  |  |  |  |  |  |
| Yes | N (%) | 15 (18) | 11 (27) | 4 (10) | 3.50 (1.07,13.71) | **0.0478*** | **0.04*** |
| Yes, Work Exposure | N (%) | 1 (1) | 1 (2) | 0 (0) | 0 | 0.991 | 0 |
| No | N (%) | 66 (80) | 29 (71) | 37 (90) | 1 |  |  |
| **Near industrial areas, farms** |  |  |  |  |  |  |  |
| Yes | N (%) | 0 (0) | 0 (0) | 0 (0) | 0 | 0 | 0 |
| Yes, Work Exposure | N (%) | 10 (2) | 6 (15) | 4 (10) | 1.25 (0.64,2.57) | 0,502 | 0,51 |
| No | N (%) | 72 (88) | 35 (85) | 37 (90) | 1 |  |  |

Significant p-value <0.05, shown in **bold***.
*Univariate non-adjusted Logistic Regression Model.
**P-value nominal significance threshold = 0.05.
***Bonferroni p-value adjusted significance threshold = 0.01.
